# Short-term effect of intravitreal dexamethasone implant in refractory diabetic macular edema

**DOI:** 10.1186/s12886-024-03341-9

**Published:** 2024-03-11

**Authors:** Jazmín Baca Moreno, David Berrones Medina, María Fernanda Rosellón-Escobar, José Gerardo García-Aguirre

**Affiliations:** 1 Retina Department, Asociación para Evitar la Ceguera en México I.A.P., Mexico City, Mexico; 2 School of Medicine and Health Sciences, Tecnológico de Monterrey, Mexico City, Mexico

**Keywords:** Diabetic macular edema, Ozurdex, Intravitreal dexamethasone

## Abstract

**Purpose:**

To evaluate the short-term effects (hours-days) of intravitreal dexamethasone implant (IDI) in eyes with diabetic macular edema (DME) refractory to anti-vascular endothelial growth factor (VEGF) injections.

**Methods:**

This was a prospective, single-arm, interventional clinical series. Eyes with DME and 3–9 injections of ranibizumab without a good response were included. Patients underwent a single IDI. Best-corrected visual acuity (BCVA) measurement, complete ophthalmic evaluation, and spectral-domain optical coherence tomography (SD-OCT) were performed at baseline, 2 h, 3 h, 24 h, 7 days, and 1 month. The main outcomes were change in central retinal thickness (CRT) on SD-OCT and BCVA.

**Results:**

Fifteen eyes of 15 patients were included. Mean CRT decreased after treatment from 515.87 µm ± 220.00 µm at baseline to 489.60 µm ± 176.53 µm after 2 h (*p* = 0.126), and 450.13 µm ± 163.43 at 24 h (*p* = 0.006). Change in BCVA was from 0.85 ± 0.44 logMAR baseline to 0.58 ± 0.37 log MAR at 1 month (*p* = 0.003).

**Conclusions:**

Eyes treated with IDI showed significant decrease in CRT detectable 1 day after injection. In some patients, the effect could be observed 3 h post-implantation.

**Trial registration:**

Clinicaltrials.gov NCT05736081. Registered 20 February 2023, Retrospectively registered.

**Supplementary Information:**

The online version contains supplementary material available at 10.1186/s12886-024-03341-9.

Diabetic macular edema (DME) is the leading cause of visual loss in patients with diabetic retinopathy (DR) [1, 2]. Global prevalence of DME is 6.8% and is higher in type 1 diabetes mellitus (T1DM) than in type 2 diabetes mellitus (T2DM) [2]. The pathophysiology of DME is an alteration in the blood-retinal barrier (BRB) induced by two complementary mechanisms: an increase in vascular endothelial growth factor (VEGF) [3] and inflammation [4, 5]. The discovery of VEGF changed the understanding and the prognosis of the disease [6]. In the past, focal/grid laser photocoagulation was the treatment of choice, but no significant vision improvement was observed in these patients until anti-VEGF agents appeared and have since become the standard treatment [6]. Nevertheless, up to 40% of patients treated with anti-VEGF have a suboptimal response, suggesting that additional factors are involved in the structural change of the BRB [7, 8].

Some of the factors that have been found elevated in the vitreous cavity of patients with DME besides VEGF are inflammatory mediators such as interleukin 6 (IL-6), interferon gamma-induced protein 10 (IP-10), membrane cofactor protein 1 (MCP-1), and platelet-derived growth factor (PDGF) [5, 9]. These factors decrease significantly with the use of intravitreal dexamethasone, proving that steroids address different targets [9]. Thus, steroids have the potential to attack additional targets beyond VEGF, and therefore be useful in cases of chronic or recalcitrant DME [10–13].

The MEAD study concluded that 0.7 mg intravitreal dexamethasone implant (IDI) (Ozurdex; Allergan Inc., Irvine, CA, USA) caused improvement in best-corrected visual acuity (BCVA) and central retinal thickness (CRT) that was significantly greater than placebo in eyes with DME [6, 11]. However, when the DRCR.net investigators compared the continuation of intravitreal ranibizumab vs switch to IDI in eyes with DME previously treated with ranibizumab, they reported no significant difference in BCVA between both groups. In the same study, the authors reported that there was a significant difference in CRT favoring IDI, suggesting that intravitreal steroids have a higher impact in drying the retina in these patients compared to antiVEGF agents [3, 14]. The aim of our study was to measure the magnitude and speed of this drying effect in eyes with refractory DME.

## Materials and methods

This was a prospective, single-arm, interventional clinical series conducted at a tertiary care center (Asociación Para Evitar la Ceguera en México, Mexico City, Mexico) between March 2018 and June 2019.

Patients older than 18 years old, with T1 or T2DM and DME involving the foveal center with CRT > 300 µm measured by OCT after at least 3 and a maximum of 9 monthly intravitreal injections of ranibizumab with or without prior panretinal photocoagulation laser treatment were included. Exclusion criteria were uncontrolled diabetes (blood glucose ≥ 250 mg/dl at any time) previous IDI, any condition precluding adequate fundus visualization, uncontrolled glaucoma, and papillary excavation ≥ 0.7. Patients that had received previous laser treatment in the macular area were excluded.

At baseline, each patient underwent a complete ophthalmologic examination, including BCVA measured with Snellen chart, intraocular pressure (IOP) measured with Goldman applanation tonometer, undilated and dilated slit-lamp biomicroscopic examination. Thirty-degree macular cube images and linear scan passing through the fovea were obtained using the Spectralis HRA + OCT (Heidelberg Engineering, Heidelberg, Germany).

All patients were treated with an IDI, and then underwent slit-lamp examination, IOP, and SD-OCT at 2 h, 3 h, 24 h, 7 days, and 1 month after the implant. BCVA and IOP were measured again at the end of the follow-up. The main outcome measure was the change in CRT on SD-OCT in response to the IDI. Secondary endpoints included BCVA and changes in IOP following intravitreal implant. Key safety variables were monitored, including ocular and systemic adverse events during the entire study duration. Descriptive statistical analysis using Microsoft Excel was performed to characterize the demographic data, the changes in CRT, BCVA, and IOP. Visual outcomes were determined by converting Snellen fractions to the logarithm of the minimum angle resolution (logMAR).

## Results

Fifteen eyes were included in the study and all the patients completed 1 month follow-up. The patient’s baseline characteristics are shown in Table 1.

**Table 1:** Baseline characteristics (*n* = 15)

**Patient no.**	**Eye**	**DM, years**	**Lens status**	**Intravitreal injections**	**PRP (sessions)**	**Blood glucose (mg/dl)**	**Retinopathy status**	**IOP (mmHg)**	**BCVA Snellen (logMAR)**	**CRT (µm)**
1	L	16	NO1NC1	4 ranibizumab, 3 aflibercept	-	179	Moderate NP	18	20/200 (1.00)	415
2	L	18	NO2NC2P2	1 bevacizumab, 3 aflibercept	-	136	Severe NP	17	20/40 (0.30)	514
3	L	15	IOL	5 aflibercept	-	139	Moderate NP	16^a^	20/150 (0.87)	430
4	R	6	IOL	4 ranibizumab	1	142	Proliferative	14^a^	CF 20 cm (1.90)	1164
5	L	26	IOL	5 ranibizumab	3	117	Proliferative	18	20/200 (1.00)	838
6	L	27	IOL	3 ranibizumab, 2 aflibercept	2	181	Proliferative	13^a^	CF 1.5 m (1.5)	617
7	R	10	IOL	2 bevacizumab, 3 ranibizumab, 2 aflibercept	2	86	Proliferative	18	20/50 (0.40)	414
8	L	10	IOL	3 bevacizumab, 4 aflibercept	-	142	Mild NP	15^a^	20/50 (0.40)	315
9	R	14	NO2NC2	8 ranibizumab, 3 aflibercept	2	110	Proliferative	12	20/200 (1.00)	452
10	R	12	IOL	3 ranibizumab, 3 aflibercept	3	118	Proliferative	14	20/200 (1.00)	489
11	R	20	NO1NC1	3 ranibizumab, 2 aflibercept	3	119	Proliferative	14	20/80 (0.60)	445
12	L	15	NO1NC2	3 ranibizumab, 6 aflibercept	4	165	Proliferative	14	20/200 (1.00)	332
13	L	13	IOL	1 bevacizumab, 1 ranibizumab, 3 aflibercept	1	108	Proliferative	15^a^	20/50 (0.40)	507
14	L	24	NO2NC2	1 ranibizumab, 3 aflibercept	3	200	Proliferative	18	20/150 (0.87)	348
15	L	6	IOL	3 bevacizumab, 6 aflibercept	4	150	Proliferative	15^a^	20/60 (0.48)	458

*F* Female, *M* Male, *DM* Diabetes mellitus, *IOP* Intraocular pressure, *BCVA* Best-corrected visual acuity, *CRT* Central retinal thickness, *PRP* Panretinal photocoagulation, *NP* Non-proliferative

^a^Previous hypotensive treatment

### Changes in macular morphology

**Fig. 1:**
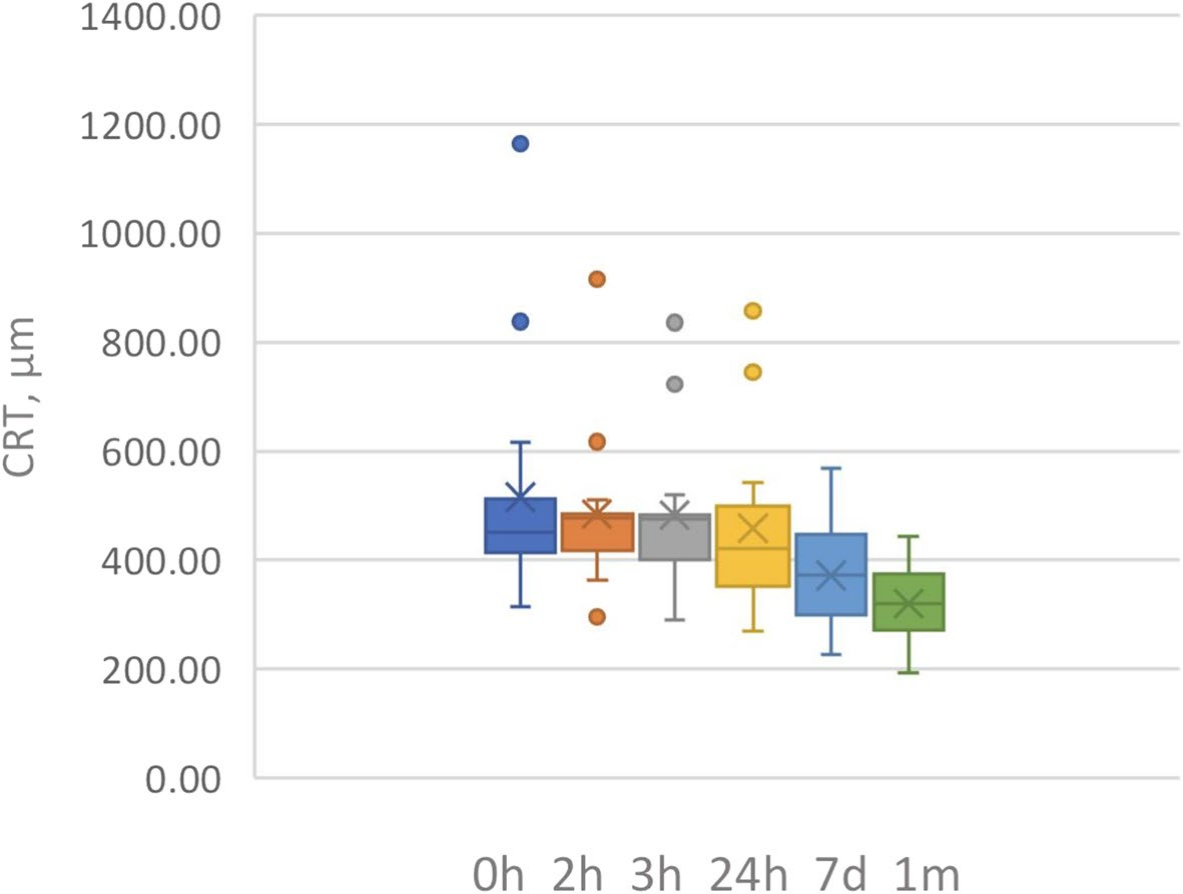
Central retinal thickness (CRT) changes

**Fig. 2:**
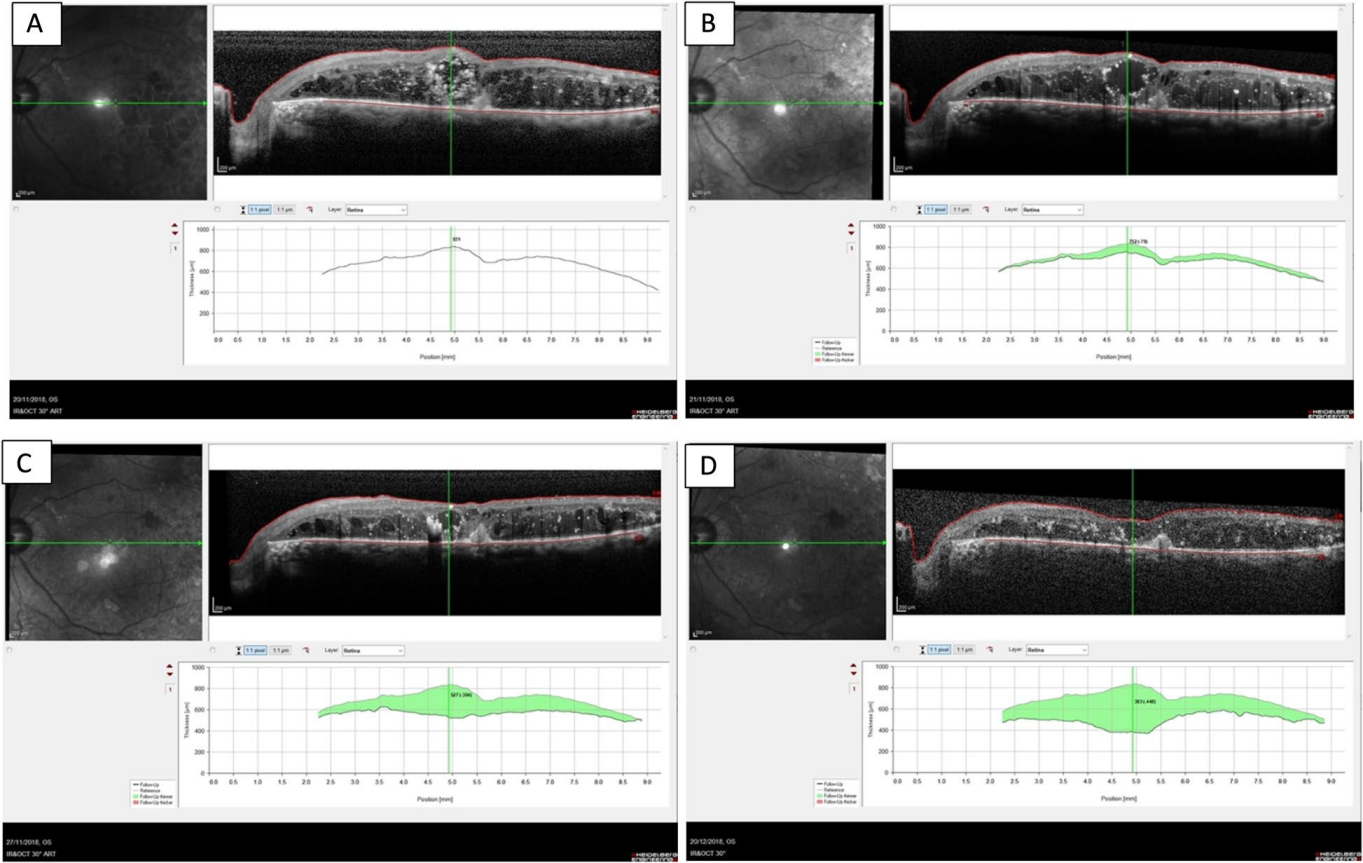
**A** Baselines OCT. **B** 1 day post injection OCT. **C** 1 week post injection OCT. **D** 1 month post injection OCT. All of the same patient

The changes in CRT are summarized in Fig. 1 and Table 2. Results indicate that CRT started to change significantly (*p* = 0.006) from 515.87 µm ± 220.00 µm at baseline to 450.13 ± 165.43 µm at 24 h. However, changes were evident in some patients since the third hour (Fig. 2). The CRT at the end of the follow-up was 319.93 ± 69.40 µm with a statistically significant difference from baseline (*p* = 0.002).

**Table 2:** CRT measures

Measure	Baseline	2 h	3 h	24 h	7 days	1 month	*P* value 1 month
CRT (µm), mean ± SD	515.87 ± 220.00	489.60 ± 176.53	487.93 ± 179.98	450.13 ± 163.43	371.87 ± 96.64	319.93 ± 69.40	< 0.002ª

*CRT* Central retinal thickness

ªCentral retinal thickness was significantly reduced after 24 h, 7 days, and 1-month post-treatment

### Changes in BCVA

BCVA had a statistically significant change (*p* = 0.003) from 0.85 ± 0.44 logMAR before IDI to 0.58 ± 0.37 logMAR at the end of the follow-up.

### Complications

An increase in IOP defined as an elevation > 10 mmHg from baseline was presented by patient number 3 and successfully managed with topical treatment. No cases of cataract progression, endophthalmitis, retinal detachment, or retinal breaks were documented during the follow up. No systemic adverse events were observed.

## Discussion

In 2010, the expected number of adults with DM worldwide by 2030 was 439 million [15]. However, the DM epidemic grew faster than predicted, and by 2019, 463 million people lived with diabetes, and the expectation for 2030 has shifted to 578 million people [16]. Nowadays, the economic burden is reported to be 10.4% of the global health expenditure [16]. Of the total patients with diabetes, one-third have diabetic retinopathy (DR) and in one-third of these, the retinopathy is vision-threatening [1, 2]. The leading cause of vision loss in DR is DME, which can occur at any stage of DR [1, 2]. It is estimated that one out of 15 people with diabetes has DME, a total of almost 31 million people worldwide [2, 16].

The main structural change accounting for DME is the disruption of the BRB, leading to hyperpermeability and vascular leakage [4, 5]. Three overlapping mechanisms have been described to explain the disruption: the first one in hyperglycemia which causes abnormalities in the polyol pathway, protein kinase C and the formation of advanced glycation end products that alter the tight junction proteins by mediators like VEGF [4–6].

The second mechanism is inflammation. Activated monocytes produce ILß, which activates NF-kB and in turn produces IL-8, MCP-1, CCL2, and TNF-alpha [4, 17]. The latter finally downregulates the production of tight junction proteins. ILß also stimulates Müller cells to produce IL-6, which increases the permeability of endothelial cells lining blood vessels, and stimulates the production of more VEGF, creating a positive feedback loop [5, 18]. Other cytokines have also shown to be higher in patients with DME, like placental growth factor (PlGF), platelet-derived growth factor (PDGF), intercellular adhesion molecule 1 (ICAM-1), interferon-inducible 10-kDa protein (IP-10), and erythropoietin (EPO) [5].

The last and most recent proposed mechanism is neurodegeneration. Multifocal electroretinogram and frequency domain optical coherence tomography have shown functional and morphological abnormalities even before observed microvascular abnormalities [19].

The first effective treatment for DME was laser photocoagulation, which achieved limited results, but it was not until 2005 that anti-VEGF agents appeared on the scene, dramatically changing the outcomes for this patient population [6, 7, 20–22]. However, although intravitreal anti-VEGF agents have much better visual outcomes than the previous treatments, continuous injections are required, and 15–40% of patients have suboptimal responses leading to a high loss-follow-up [6–8, 23–26]. One theory of this suboptimal response with anti-VEGF agents is that their intravitreal concentration progressively decreases between injections.5 Additionally, anti-VEGF agents decrease VEGF levels in a very potent and efficient fashion but do not have an impact on other cytokines that have been implicated in the pathophysiology of DME, such as IL-6, IP-10, MCP-1, PDGF-AA [9]. On the other hand, steroids have proven to significantly reduce the levels of these other cytokines, and also VEGF, and may therefore prove useful in cases of refractory DME [9–13].

Despite this theoretical benefit of steroids, the group that received intravitreal triamcinolone in the DRCR.net Protocol I did not achieve significant visual gains when compared to the intravitreal anti-VEGF agent [27, 28]. This outcome was explained in part because 59% of the patients developed a cataract. The results appeared comparable when comparing triamcinolone to ranibizumab just in pseudophakic patients [28]. Another adverse effect of steroids is the increase in IOP which in this protocol was defined as an IOP elevation > 10 mmHg from baseline, IOP > 30 mmHg, or the initiation of IOP-lowering medication, and occurred in 50% of the patients in the triamcinolone group [28].

Different studies reported improvements in CRT and BCVA using IDI in VEGF-refractory DME, with cataracts, and increased IOP as adverse effects [10, 11]. However, Protocol U, a randomized controlled trial, found that IDI only improves CRT but not BCVA [3, 29]. One of the possible reasons for this seemingly paradoxical effect is the occurrence of other factors such as macular ischemia or atrophy that led to irreversible foveal damage [14].

The outcomes of protocol U were confirmed by a meta-analysis, with a statistical significance in the mean reduction of CRT at 6 months but not at 12 months [30]. On the other hand, a different meta-analysis did show a significant improvement of 4 lines in BCVA in the IDI group compared to the anti-VEGF group [12].

Although there are many long-term studies of IDI for DME, its short-term effect has only been evaluated in a handful of studies [12, 31–33]. Three different Italian groups made each a prospective, single-center, single-arm, interventional case series (Table 3). The number of eyes included in these series were 8–23, with an average age from 65 to 68.7 years. The three-case series were included both naive and chronic patients. All series excluded patients with uncontrolled glaucoma, elevated IOP, and inadequate fundus visualization. The follow up evaluations were made within the first hours, up to 1 or 3 months. At baseline and in the follow-up, all patients underwent a complete ophthalmologic examination and OCT. All patients were treated with a single IDI [31–33]. The mean BCVA change in the group that end the follow-up at 1 month was 0.14 logMAR vs 1.53 logMAR in both groups that continued the follow-up to 3 months. Mean CRT change range from -183.9 µm in a study that followed patients for 1 month, to -255 µm in a study that followed patients for 3 months.

**Table 3:** Case series comparison

	**Veritti, 2017** [31]	**Giuseppe, 2018** [32]	**Minnella, 2019** [33]	**Current study**
Eyes	23	18	8	15
Age (years)	67.7	65	68.7	68
Glucose mg/dL	-	< 250	-	< 250
HbA1c %	< 7.3	< 9	-	-
Baseline lens	-	-	7 phakic	6 phakic
Naïve/chronic	9/14	12/6	3/5	0/23
Previous tx	No steroids in the last 6 mo	None in the last 3 mo	None in the last 6 mo	At least 3 continuous antiVEGF
OCT	Cirrus, Zeiss	Cirrus, Zeiss	Heidelberg, Spectralis	Heidelberg, Spectralis
Evaluation				
Hours	-	1,3	3	2,3
Days	1,2,3,7,14,21	3,7	1,7	1,7
Months	1,2,3	1	1,3	1
BCVA mean change	1.52 logMAR at 3 mo	0.14 logMAR at 1 mo	1.52 logMAR at 3 mo	0.27 logMAR at 1 mo
CRT mean change (µm)	-256	-225	-183.9	-195.93

*BCVA* Best-corrected visual acuity, *CRT* Central retinal thickness

In our study, we aimed to prospectively investigate the short-term effects of IDI on macular morphology and visual function in patients with at least 3 consecutive antiVEGF injections, with any baseline BCVA for 1 month. The average CRT reduction observed in our patients was 195.92 µm at 1 month. Some patients had an evident reduction in CRT since the third-hour post-injection, but the reduction achieved statistical significance until the 24 h follow-up. This effect was also observed in the two previous series that started their follow-up within hours [32, 33]. Minnella, 2019, also compared naïve and chronic patients and found that CRT reduction was greater in chronic patients [33].

One difference between that series and ours is that they included 33% of chronic patients, while we had 100% [32]. Overall, gains in BCVA were greater in the groups that continued the follow-up for 3 months [31, 33]. However, there was no correlation between BCVA and the mean CRT reduction among the four series [31–33].

The main adverse effect reported in these case series was increase in IOP, managed with topical treatment and no statistical significance [31–33]. Neither study had cataract progression, endophthalmitis, retinal detachment, or retinal breaks. No systemic adverse effects were observed [31–33]. The major limitation in all studies, including ours, are the small number of eyes and the lack of control arm.

In conclusion, a single IDI in eyes with treatment resistant DME causes a swift and significant reduction in CRT that is detectable at 3 h, and statistically significant at 24 h post-implantation. This effect may be explained by the inhibition of additional inflammatory factors beyond VEGF that also play a role in the pathogenesis of DMR. However, further research is needed to clarify if this option has better outcomes when compared to antiVEGF monotherapy.

### Supplementary Information

**Supplementary material 1.**

## Data Availability

Data is provided with the manuscript or supplementary information files.

## References

[1] Holekamp N (2016). Overview of diabetic macular edema. Am J Manag Care.

[2] Tan G, Cheung N, Simó R, Cheung G, YinWong T (2017). Diabetes macular oedema. Lancet Diabetes Endocrinol.

[3] Bressler NM (2018). Early response to anti-vascular endothelial growth factor and two-year outcomes among eyes with diabetic macular edema in protocol T. Am J Ophthalmol.

[4] Tang J, Kern T (2011). Inflammation in diabetic retinopathy. Prog Retin Eye Res.

[5] Noma H, Yasuda K, Shimura M (2021). Involvement of cytokines in the pathogenesis of diabetic macular edema. Int J Mol Sci.

[6] Wallash J, Gallemore R (2021). Anti-VEGF-resistant retinal diseases: a review of the latest treatment options. Cells.

[7] Sun JK, Jampol LM (2019). The Diabetic Retinopathy Clinical Research Network (DRCR.net) and its contributions to the treatment of diabetic retinopathy. Ophthalmic Res.

[8] Bressler S (2016). Persistent macular thickening after ranibizumab treatment for diabetic macular edema with vision impairment. JAMA Ophthalmol.

[9] Sohn H (2011). Changes in aqueous concentrations of various cytokines after intravitreal triamcinolone versus bevacizumab for diabetic macular edema. Am J Ophthalmol.

[10] Chronopoulos A, Chronopoulos P, Ashurov A, Korb C, Pfeiffer N, Hattenbach LO. Switching to intravitreal fluocinolone acetonide implant for refractory diabetic macular edema: 12- and 24-month results. Eur J Ophthalmol. 2022;32(1):443–9. 10.1177/1120672121992982. 10.1177/1120672121992982 33601897

[11] Boyer D, Hee Y, Belfort R, Hashad Y, Whitcup S (2014). Three-year, randomized, sham-controlled trial of dexamethasone intravitreal implant in patients with diabetic macular edema. Ophthalmology.

[12] Zainab K, Kuriakose R, Khan M, Chin E, Almeida D (2017). Efficacy of the intravitreal sustained-release dexamethasone implant for diabetic macular edema refractory to anti-vascular endothelial growth factor therapy: meta- analysis and clinical implications. Ophthalmic Surg Lasers Imaging Retina.

[13] Maturi RK (2018). Effect of adding dexamethasone to continued ranibizumab treatment in patients with persistent diabetic macular edema: a DRCR network phase 2 randomized clinical trial. JAMA Ophthalmol.

[14] Nunes S, Pereira I, Santos A, Bernardes R, Cunha-Vaz J (2010). Central retinal thickness measured with HD.OCT shows a weak correlation with visual acuity in eyes with CSME. Br J Ophthalmol.

[15] Herman W, Dagogo-Jack S (2017). The global burden of diabetes: an overview. Diabetes mellitus in developing countries and underserved communities.

[16] Karuranga S, Malanda B, Saeedi P, Salpea P, eds. IDF atlas ninth edition: Brussels: International diabetes federation; 2019. Available at: https://diabetesatlas.org/upload/resources/material/20200302_133351_IDFATLAS9e-final-web.pdf.

[17] Rangasamy S, McGuire P, Franco C, Monickaraj F, Oruganti S, Das A (2014). Chemokine mediated monocyte trafficking into the retina: role of inflammation in alteration of the blood-retinal barrier in diabetic retinopathy. PLoS One.

[18] Liu X (2015). IL-1B induces IL-6 production in retinal Müller cells predominantly through the activation of the p38 MAPK/NF-kB signaling pathway. Exp Cell Res.

[19] Simó R, Hernández C (2014). Neurodegeneration in the diabetic eye: new insights and therapeutic perspectives. Trends Endocrinol Metab.

[20] Early Treatment Diabetic Retinopathy Study Research Group (1985). Photocoagulation for diabetic macular edema. Arch Ophthalmol.

[21] Kim L, D’Amore PA (2012). A brief history of anti-VEGF for the treatment of ocular angiogenesis. Am J Pathol.

[22] Hoffmann-La Roche F (2012). FDA approves Lucentis (ranibizumab injection) for treatment of diabetic macular edema.

[23] Diabetic Retinopathy Clinical Research Network (2010). Randomized trial evaluating ranibizumab plus prompt or deferred laser or triamcinolone plus prompt laser for diabetic macular edema. Ophthalmology.

[24] Nguyen Q (2012). Ranibizumab for diabetic macular edema: results from 2 phase III randomized trials: RISE and RIDE. Ophthalmology.

[25] Brown E (2013). Long-term outcomes of ranibizumab therapy for diabetic macular edema: the 36-month results from two phase III trials: RISE and RIDE. Ophthalmology.

[26] Chatziralli I, Loewenstein A (2021). Intravitreal anti-vascular endothelial growth factor agents for the treatment of diabetic retinopathy: a review of the literature. Pharmaceutics.

[27] González V (2016). Early long-term response to anti-vascular endothelial growth factor therapy in diabetic macular edema: analysis of protocol I data. Am J Ophthalmol.

[28] Elman MJ (2010). Randomized trial evaluating ranibizumab plus prompt or deferred laser or triamcinolone plus prompt laser for diabetic macular edema. Ophthalmology.

[29] Maturi R. Digging deeper into protocol U. Review of Ophthalmology, Jobson Optical Group, Medical Information; 2018. Available at: https://www.reviewofophthalmology.com/article/digging-deeper-into-protocol-u.

[30] He Y, Ren XJ, Hu BJ, Lam WC, Li XR (2018). A meta-analysis of the effect of dexamethasone intravitreal implant versus intravitreal anti-vascular endothelial growth factor treatment for diabetic macular edema. BMC Ophthalmol.

[31] Giudice G, Avarello A, Campana G, Galan A (2018). Rapid response to dexamethasone implant in diabetic macular edema. Eur J Opthalmol.

[32] Veritti D, Sarao V, Galiazzo F, Lanzetta P (2017). Early effects of dexamethasone implant on macular morphology and visual function in patients with diabetic macular edema. Ophthalmologica.

[33] Minnella A, Federici M, Pagliei V, Lanza A, Gambini G, Caputo C (2019). Short- term assessment of intravitreal dexamethasone implant using enhanced depth image optical coherence tomography and optical coherence tomography angiography in patients with retinal vascular diseases. Adv Ther.

